# Stringent Regulations of Oocyte Donation Among Jewish Women in Israel: Characteristics and Outcomes of the National Oocyte Donation Program in One Central IVF Unit

**DOI:** 10.1007/s10943-024-02200-7

**Published:** 2024-12-09

**Authors:** Livia Preisler, Nivin Samara, Yael Kalma, Tali Arad, Asnat Groutz, Foad Azem, Hadar Amir

**Affiliations:** Racine IVF Unit, Affiliated to the Sackler Faculty of Medicine, Tel Aviv Sourasky Medical Center, Fertility Institute, Lis Maternity Hospital, Tel Aviv University, 6 Weizmann Street, Tel Aviv, Israel

**Keywords:** Oocyte donation, Oocyte donors, Oocyte recipients, Financially compensated volunteers, Israeli law, Outcomes

## Abstract

On September 5, 2010, the Israeli Parliament passed a law that allows Israeli female residents to donate their oocytes to infertile Israeli female residents. This law includes unique restrictions that do not exist in other countries. Our aim was to characterize Israeli oocyte donors and recipients and the outcomes of the oocyte donation program as regulated by national law. This retrospective study included 26 financially compensated volunteer donors (mean age 29 ± 3.52 years) and 69 recipients (mean age 44.6 ± 3.53 years) who underwent 30 intracytoplasmic sperm injection cycles and 166 embryo transfers (ETs) in our unit between March 2016 and November 2020. Stringent legal caveats unique to Israel (e.g., Jewish/Moslem donor only to Jewish/Moslem recipient, only unmarried donor, eggs in one cycle restricted to ≤ 3 recipients, donated sperm only from non-Jewish donors, and more) were meticulously applied. Sociodemographic characterizations of donors and recipients were reviewed, and pregnancy and obstetric outcomes were determined. Variables that were significant in achieving live births among the recipients were examined. Twenty-five donors and all 69 recipients were Jewish, and most were unmarried and childless. The main indication for seeking egg donation was age ≥ 40 years/perimenopause (80%). One-half of the recipients used donor sperm and one-half used partner sperm. The pregnancy, clinical pregnancy, live birth, and miscarriage rates were 28.6%, 19.2%, 18.2%, and 2.8%. The live birth rate was negatively associated with multiple ETs. Maternal complications included hypertensive disorders of pregnancy (18.2%), gestational diabetes mellitus (32.3%), and caesarean sections (78.8%). There were no adverse neonatal outcomes. In conclusion, few young women are interested in donating oocytes in Israel. Pregnancy and live birth rates are lower than published values in other egg donation programs.

## Introduction

Oocyte donation (OD) has greatly enhanced our ability to treat infertility for traditional indications, including diminished ovarian reserve, primary ovarian insufficiency, poor oocyte quality, genetic problems, and more recently perimenopause and menopause patients, the latter in increasing the request for donated oocytes (Sauer & Kavic, 2006).

Family law in Israel is mainly governed by religious law (be it Orthodox-Jewish, Sharia-Muslim, Druze or Christian law) and adjudicated by the religious courts. Therefore, it is important to familiarize oneself with some religious laws related to the issues raised in this paper. The rabbis of Israel support fertility treatments. The Jewish perspective towards procreation originally comes from the first commandment in the Jewish Torah (Bible) that Adam received from God to “be fruitful and multiply” (Genesis, 1:18). Thus, as long as no prohibition is violated, any method of procreation may be used to produce pregnancy.

Nevertheless, many Jewish authorities’ express concerns over the unclear religious status of children born via ED. Jewish identity is established at birth. A child born to a Jewish mother is fully Jewish regardless of the biological father’s religious identity. A child born to a non-Jewish mother acquires a Jewish identity only by a halakhic conversion performed by a properly constituted rabbinic court. In the case of ED, a problem arises: who should be considered the mother? The oocyte donor or the woman in whose uterus the embryo develops? In a case where one of the women is Jewish and the other is not, this problem will arise. This is because according to Jewish law, a child’s religious status is determined by his mother. Early rabbinic verdicts determine that the child is related to the woman who finished forming it- she who gave birth. However, recently, following modern medical discoveries, other senior religious authorities opined that it is the genetic mother who is the halakhic mother. Being that this issue is not settled in the Jewish community, the general practice requires that the child undergo a religious conversion when the oocyte donor and the gestational mother are not both Jewish (Grazi & Wolowelsky, 2014; Gruenbaum et al., 2011). Thus, when both are Jewish, this problem does not arise.

Regarding the sperm donor’s identity, all rabbis are unanimously against using sperm from a Jewish donor. Some of them maintain that a married woman who was impregnated by a Jewish donor is to be considered as having committed adultery and hence must not remain married to her husband. An additional concern relates to the potential of an unwitting marital relationship between siblings of the same donor; the chances of such incestuous relations are almost null when sperm is imported from non-Jews abroad. Therefore, all rabbis recommend avoiding the purchase of semen from Israel (Bokek-Cohen, 2016).

Another problem that emanates from ED is that of a child’s mamzer status. According to Jewish law, a mamzer is the result of an adulterous relationship- a child born out of wedlock, i.e. as a result of an affair the wife had with another man while she was married. A mamzer is also a child who is a result of incest. Mamzer status may be similar to that of a bastard in western society, but it does not quite hold identical stigma. A mamzer must obey harsh restrictions when marrying within the Jewish community- they can only marry another mamzer or a convert. This applies ten generations forward (most scholars take that to mean for eternity, but others take it literally). If a married woman was to donate eggs that would later be fertilized by sperm that does not belong to her husband, the child born as a result would be considered a mamzer (Schenker, 2005).

As for the Sunni Islamic population, other religious limitations exist regarding ED. Fertility treatments are permissible only when they do not entail any form of third-party donation or involvement (of sperm, eggs, embryos, or uterus). All third-party gamete or embryo donations are forbidden. This prohibition was intended to safeguard paternity and the institution of the family, one of the five goals of Islamic law. Any such third-party interference could affect the lineage, and in turn, the legal relationship between the children and the parents under Islamic Sharia’h. Because Islamic law maintains a strict prohibition against sexual relations outside marriage and against *zina* (adultery), it considers third-party donated gametes *zina*, although donating gametes does not involve any physical contact between donor and recipient. In addition, third-party donation may incur the risk of incest between two donor offspring who may later meet without knowledge of their common lineage and marry each other (Bokek-Cohen et al., 2022).

Until 2010 Israeli law stated that only women who are undergoing fertility treatment can donate ova, and that unpaid donors can do so for altruistic reasons alone (the “shared” OD program) (Levran et al., 1996; Rabinerson et al., 2002). These restrictions had created a great scarcity of available eggs because only a limited number of surplus eggs were left for donation (Gruenbaum et al., 2011). To at least partially overcome this, the law was amended in 2005 to approve ODs from overseas donors (the “tourism” OD program) (Ministry of Health, State of Israel, 2005).

Imported eggs are not suitable for all Israeli women for a number of reasons. First and foremost, it was demonstrated that the most obvious criterion a recipient women/couple would look for in a prospective egg donor is her/their race, ethnicity, and religion to match their own family heritage (Heng, 2007). In Israel, where a significant portion of the population is observant, whether it is Judaism, Islam (mostly Sunni), Christianity, or other, there are women/couples for whom the religion of the donor woman is critical. Importantly, Jewish law states that only the offspring of a Jewish mother are considered as having been born as a Jew (Ezra 10:3). As a result, many rabbis decree that any child born from oocytes derived from non-Jewish donors must undergo formal religious conversion shortly after birth. The rabbinical definition of a mamzer is the offspring of a married woman who is impregnated by a man who is not her husband, therefore, the oocyte donor must be unmarried in order to prevent mamzerut (Schenker, 2005). According to Sunni Islam law, OD is generally forbidden (Chien, 2020) and an exceptional approval of the clergy is required. Another reason is the recipients’ wish for maximal physical resemblance to their donor (Klock & Greenfeld, 2004). The costs of procuring oocytes from abroad is often prohibitive and place a huge financial burden on women/couples contemplating OD (Gruenbaum et al., 2011; Nahman, 2011). At the same time, some recipient women/couples may believe that Israel’s restrictive donor selection process is reassuring and that it will contribute to their sense of security. For these and other reasons, Israeli women who were interested in eggs from Israeli donors have been left without an adequate solution.

In 2010 the Israeli ED law was changed (Ministry of Health, State of Israel, 2010). The law allows women between the ages of 21 and 35 years to donate their eggs for a value set by the Ministry of Health (MOH). The law allows infertile women between the ages of 18 and 54 years to request OD, which will be partly covered by national insurance plans. This requires that both the donor and the recipient will be Israeli residents and that the donor’s identity will be kept confidential. The Israeli law states that the donor and the recipient must share the same religion and that the religion of the donor is to be made known to the future parent/s. In addition, the oocyte donor must be an unmarried woman. The law also increases the extent to which the government oversees and regulates the process, such as limiting a woman to a maximum of three donations with a six-month interval between each intervention and limiting the number of eggs retrieved in one cycle to no more than three per donor. Finally, the ovum pick-up and subsequent fertilization must be performed in Israel.

Previous studies that reported OD outcomes in Israel described either the “shared" (Levran et al., 1996; Rabinerson et al., 2002; Yaron et al., 1998) or the "tourism" OD (Sheffer-Mimouni et al., 2002; Yerushalmi et al., 2021) programs. However, the former also included in vitro fertilization (IVF) patients as donors and the second also included various OD programs with non-Israeli donors. The present study is, therefore, the first to characterize the Israeli financially compensated volunteer oocyte donors and their recipients and to determine the OD program outcomes under the auspices of the revised national law.

## Methods

### Study Population and Participant Recruitment

This retrospective study was performed between March 2016 and November 2020 at the IVF Unit, Fertility Institute, in the Tel Aviv Sourasky Medical Center, a tertiary university-affiliated medical center. The OD program was initiated in our IVF Unit in 2016 (6 years after the revised law was enacted). The data on all ODs and the characteristics of the donors and recipients, all of whom were residents of Israel, were retrieved and analyzed. All donations were completely anonymous. All donors were approved for participation in the OD program by a committee comprised of six members, including a head of a non-gynecological department, fertility specialist, psychologist, social worker, lawyer, and Rabbi. All these professionals are required by law to attend the committee.

#### Donors

The exclusion criteria for donors were: (1) low ovarian reserve defined as basal follicle-stimulating hormone (FSH) > 10. (2) candidates found by genetic screening to be carriers of genetic diseases. (3) an antral follicle count (AFC) < 12. The law allows a division of oocytes from one donor to a maximum of three recipients. The hospital receives full coverage of the procedure only when three women receive donations from one woman. In our unit, we have committed to at least 4 mature oocytes for each recipient. Therefore, donors with AFC < 12 were excluded. Twenty-six suitable ovum donors were included in this study. Their ages ranged between 21 and 35 years, and they were comprised of financially compensated volunteers who were not undergoing fertility treatments, and unmarried. Twenty-five were Jewish and one did not identify with any religion. They received a detailed explanation of the procedure for donating eggs, including hormonal treatment, oocyte retrieval, complications, and associated risks. They all signed the MOH’s “Egg Donor’s Consent Form for the Retrieval and Donation of Eggs”. In addition, their signed consent was required for the inclusion of their details in the database of the MOH and Newborn Registry. They then underwent medical examinations, including a physical examination, laboratory tests, genetic screening, a PAP smear, breast examination and an assessment by a clinical psychologist. Prior to providing the egg donation, the approval committee ruled that the consent to donate the eggs was granted lucidly, out of free will, and not from family, social, financial, or any other pressures. After the retrieval, each donor was informed of the number and quality of the retrieved eggs. As of 2021, each donor was paid 20,462 shekels (approximately $6500) per retrieval cycle. One-half of the payment was provided by the ovum recipient and the rest by the recipient’s Health Maintenance Organization. Twenty-two donors donated once and four donated twice with a six-month interval. The eggs retrieved in one cycle were allocated to no more than three women.

#### Recipients

Sixty-nine ovum recipients were included. They were comprised of infertile women between 18 and 54 years of age. All recipients signed the MOH’s “Application Form for Recipient of Egg Donation” and a second consent for the inclusion of her details in the MOH database and Newborn Registry. They underwent comprehensive medical examinations, as detailed for the donors. The law does not mention a recipient’s alcohol or drug use as a contraindication for receiving OD. When the sperm donation was anonymous, the law requires that only the sperm of a non-Jewish donor was used. The commitment for each recipient was a minimum of 4 mature oocytes. A recipient who subsequently did not undergo embryo transfer (ET) was excluded from the study.

### Data Retrieval

All relevant data were collected from the computerized hospital database ("Evepro" and "Chameleon"). The data recorded in the electronic charts included clinical and demographic details of the oocyte donors [age, marital status, ethnicity, religion, number of children, employment, medical history, chronic medications, weight, height, body mass index (BMI), smoking status, alcohol consumption, drug abuse, basal FSH levels, thyroid-stimulating hormone (TSH) levels, prolactin levels, AFC], clinical and demographic details of the oocyte recipients [age, marital status, ethnicity, number of children, employment, medical history, chronic medications, weight, height, BMI, smoking status, alcohol consumption, drug abuse, infertility reason, sperm source], assisted reproductive technology (ART) details and outcomes [number of ovum pick-up (OPU) cycles per oocyte donors, ovarian stimulation duration, total FSH levels, peak serum estradiol, number of retrieved oocytes, number of metaphase II (MII) oocytes, number of 2 pronuclei (2PNs), number of usable embryos (either transferred or cryopreserved), ET protocol type, endometrial thickness before ET, day the embryos were transferred, and number of embryos transferred], and pregnancy and obstetric outcomes as detailed below.

Serum beta human chorionic gonadotropin (β-hCG) testing with a result > 25 IU/I was considered positive for pregnancy (Lan et al., 2021; Urbancsek et al., 2002). A clinical pregnancy was confirmed by the observation of an embryo pulse on transvaginal ultrasound scanning at 6 to 12 weeks of gestation. Early miscarriage was diagnosed when a previously positive pregnancy test became negative before ultrasonographic detection of an embryonic pulse in the sixth week of pregnancy or later (Hoffman et al., 2022). Miscarriage was defined as a loss of a clinical pregnancy before 12 full weeks of gestation. Live birth was defined as a live neonate born after 24 weeks of gestation. Pregnancy, clinical pregnancy, and live birth rates were on a “per embryo transfer” basis. Early miscarriage and miscarriage rates were calculated per pregnancy.

The examined maternal outcomes were hypertensive disorders of pregnancy (HDP) that included pregnancy-induced hypertension (PIH, defined as systolic blood pressure > 140 mm Hg and/or diastolic blood pressure > 90 mm Hg after 20 weeks’ gestation) or preeclampsia (defined by hypertension associated with proteinuria > 300 mg/24 h), gestational diabetes mellitus (GDM, defined as having one or more abnormal values of a 3-h 100 g oral glucose tolerance test between 24 and 28 weeks of gestation, with the cut-off points of 95 mg/dl for fasting, 180 mg/dl for 1 h, 155 mg/dl for 2 h and 140 mg/ for 3 h), and mode of delivery [spontaneous, operative vaginal and caesarean section (CS)]. The perinatal outcomes in the present study were preterm birth (PTB, live birth before 37 gestational weeks), large for gestational age (LGA, birth weight above the 90th percentile for gestational age by infant sex), small for gestational age (SGA, birth weight below the 10th percentile for gestational age by infant sex), appropriate for gestational age (AGA, birth weight between the 10th and 90th percentile for gestational age by infant sex), and Apgar score < 7 at 1 and 5 min.

### Ovarian Stimulation, Fertilization, Embryo Culture and Embryo Transfer

Controlled ovarian stimulation of all donors was carried out by gonadotropin releasing hormone (GnRH) antagonist protocols (Amir et al., 2019). Ovulation was triggered with 0.2 mg of triptorelin (Decapeptyl; Ferring Pharmaceuticals) when at least three follicles achieved a diameter of 18 mm. Ovum pick-up was performed 36 h later, and embryologists determined the total number of oocytes retrieved per cycle.

All embryos were fertilized by intracytoplasmic sperm injection (ICSI) in which cumulus cells were removed 2–3 h after retrieval and oocytes maturity was determined. Only MII oocytes were considered mature. All embryos were incubated in the integrated EmbryoScope™ time-lapse monitoring system (EmbryoScope™; UnisenseFertiliTech A/S, Aarhus, Denmark, Vitrolyfe) from the time of fertilization until ET or freezing. ET or cryopreservation was carried out between two to six days following oocyte retrieval. In the cases of frozen ET (FET), endometrial preparation was performed with modified natural or artificial (hormonally substituted) cycle protocols (Yarali et al., 2016). Luteal support with progestin supplement (in various regimens) was continued until a negative β-hCG or the ninth week of pregnancy. Serum β-hCG levels were measured on day 14 after ET.

A single treatment cycle was defined by oocyte retrieval and all transfers (i.e., fresh and frozen-thawed) derived from that ovarian stimulation. One complete treatment cycle was defined by a treatment cycle that achieved a live birth or in which all embryos were transferred but failed to achieve a live birth. Only the first delivery of each recipient was considered in the analysis.

### Statistical Analysis

Data were analyzed with SPSS, version 27.0 (SPSS, Inc., Chicago, IL, USA). They are presented as mean ± standard deviation (SD) for continuous variables and as proportion for categoric variables. The association between donor and recipient age, number of mature oocytes per recipient, sperm source, number of frozen embryos, and number of ET cycles, and the outcome of live birth was assessed with the use of generalized linear mixed models which take into account the repeated measures for donors. A *p* value of < 0.05 was considered significant.

## Results

### Clinical Characteristics of the Study Participants

During the 5-year study period, 26 anonymous women donated oocytes to 69 infertile females of whom 65 underwent ET and were included in the present study. The sociodemographic and clinical characteristics of the entire cohort are detailed in Table 1. Twenty-five donors were Jewish (one egg donor was defined irreligious as her father is Jewish but her mother is not) and most were Ashkenazi (53.8%), single (80.8%), childless (88.5%), and currently employed (84.6%). The donors’ mean age and BMI were 29 ± 3.52 years and 24.2 ± 5.12 kg/m^2^, respectively. The ovarian reserve markers, FSH and AFC, of all donors were within the normal range (6.8 ± 2.12 mIU/mL and 17.1 ± 5.61, respectively).

**Table 1:** Demographics of Israeli oocyte recipients and volunteer oocyte donors

Donors (n = 26)	
Age (y), mean (SD)	29 (3.52)
BMI (kg/m^2^), mean (SD)	24.2 (5.12)
Behaviors (n, %)	
*Smoking*	
Yes	8 (30.8)
No	18 (69.2)
*Alcohol*	
Yes	0 (0)
No	26 (100)
*Drugs*	
Yes	2 (7.7)
No	24 (92.3)
*Religion (n, %)*	
Jewish	25 (96.2)
Irreligious	1 (3.8)
*Ethnicity (n, %)*	
Ashkenazi	14 (53.8)
Sephardic	4 (15.4)
Mixed	8 (30.8)
*Marital status (n, %)*	
Single	21 (80.8)
Divorced	5 (19.2)
*Children, n (%)*	
Yes	3 (11.5)
No	23 (88.5)
*Employment*	
Employed	22 (84.6)
Unemployed	0 (0)
Student	4 (15.4)
Day 3 FSH (mIU/mL), mean (SD)	6.8 (2.12)
TSH (μIU/mL), mean (SD)	1.9 (0.8)
Prolactin (mIU/L), mean (SD)	202.8 (99.8)
AFC (total number), mean (SD)	17.1 (5.61)
Recipients (n = 65)	
Age (y), mean (SD)	44.6 (3.53)
BMI (kg/m^2^), mean (SD)	25.7 (6.39)

*Chronic disease (n, %)*	
Hypothyroidism	9 (13.8)
DM type 1	2 (3.1)
DM type 2	1 (1.5)
Hypertension	3 (4.6)
*Behaviors (n, %)*	
Smoking	
Yes	10 (15.4)
No	55 (84.6)
Alcohol	
Yes	1 (1.5)
No	64 (98.5)
Drugs	
Yes	1 (1.5)
No	64 (98.5)
*Religion (n, %)*	
Jewish	65 (100)
Other	0 (0)
*Marital status (n, %)*	
Single	34 (52.3)
Married	26 (40)
Divorced	5 (7.7)
*Children, n (%)*	
Yes	17 (26.2)
No	48 (73.8)
*Cause of OD, n (%)*	
Perimenopause	52 (80)
Menopause	9 (13.8)
POI/POF	3 (4.6)
Genetic problem	1 (1.5)
*Sperm source, n (%)*	
Partner	32 (49.2)
Donor	33 (50.8)
Age of recipient's partner (y), mean (SD)	45 (6.6)

Values are presented as mean (SD) or number (%)

BMI, body mass index; FSH, follicle-stimulating hormone; TSH, thyroid stimulating hormone; AFC, antral follicle count; DM, diabetes mellitus; OD, oocyte donation; POI, premature ovarian insufficiency; POF, premature ovarian failure; SD, standard deviation

Standard reference ranges: FSH: 1–9.2 mIU/mL; TSH: 0.5–4.8 μIU/mL; Prolactin: 108.78–557.13 mIU/L

All of the recipients were Jewish, 34 (52.3%) were single, 26 (40%) were married, and 5 (7.7%) were divorced. Forty-eight (73.8%) were childless. The recipient’s mean age and BMI were 44.6 ± 3.53 years and 25.7 ± 6.39 kg/m^2^, respectively. One recipient reported occasional use of alcohol and drugs. The indications for using donor oocytes were as follows: age ≥ 40 years /perimenopause (n = 52, 80%), menopause (n = 9, 13.8%), premature ovarian insufficiency/premature ovarian failure (n = 3, 4.6%), and a genetic problem (n = 1, 1.5%). Thirty-two (50%) of the women used partner sperm for ovum fertilization, and half used donor sperm.

### ART Data and Outcomes

The donors underwent a total of 30 OPU cycles: specifically, seven in 2016; five in 2017; six in 2018; five in 2019; and seven in 2020. Four donors completed two donation cycles and the rest underwent a single cycle. The ART data and outcomes are summarized in Table 2. The mean number of FSH stimulation days and the amount of FSH used were 10.6 ± 1.39 and 2940 ± 646.79 mIU/mL, respectively. The mean peak estradiol levels, number of retrieved oocytes, number of MII oocytes, and oocyte maturity rates were 2646 ± 1673.4 pg/mL, 23.6 ± 14.81, 19.1 ± 13.19, and 80.9 ± 14.75%, respectively. The mean number of MII oocytes per recipient from their respective donors was 7.7 ± 4.28. The ICSI fertilization rate was 83.7 ± 15.89%, and the mean number of cryopreserved embryos was 4.6 ± 1.87.

**Table 2:** Cycle characteristics of Israeli oocyte recipients and volunteer oocyte donors

Donors (n = 26)	
Number of OPU cycles	30
OPU cycles per patient, mean (SD)	1.2 (0.4)
*No. of OPU cycles per patient, n (%)*	
1	22 (84.6)
2	4 (15.4)
*No. of recipients per OPU cycle, n (%)*	
1	4 (13.3)
2	6 (20)
3	20 (66.7)
Ovarian stimulation duration (days), mean (SD)	10.6 (1.39)
FSH total dose (mIU/mL), mean (SD)	2940 (646.79)
Peak E2 (pg/mL), mean (SD)	2646 (1673.4)
Oocytes retrieved (n), mean (SD)	23.6 (14.81)
MII oocytes (n), mean (SD)	19.1 (13.19)
Maturity rate, % (SD)	80.9 (14.75)

Recipients (n = 69)	
Donated MII oocytes per recipient (n), mean (SD)	7.7 (4.28)
2PN embryos, mean (SD)	6.4 (3.54)
Fertilization rate, % (SD)	83.7 (15.89)
Total number of embryos, mean (SD)	4.6 (1.87)
Number of ET cycles	**166**
ET cycles per patient, mean (SD)	2.3 (1.25)
*ET protocol type, n (%)*	
Fresh ET	5 (3)
Modified natural FET	154 (92.8)
Hormonally substituted FET	7 (4.2)
Endometrial thickness (mm), mean (SD)	8.6 (1.38)
Number of embryos transferred per transfer, mean (SD)	1.1 (0.31)
*Number of embryos transferred, n (%)*	
1	148 (89.2)
2	18 (10.8)
*Day of ET, n (%)*	
Day 2 or 3	151 (91)
Day 5 or 6	14 (8.4)
Day 3 and 5	1 (0.6)

Pregnancy results are calculated per number of embryo transfers given in bold

Values are presented as mean (SD) or number (%)

OPU, Ovum pick-up; FSH, Follicle-stimulating hormone; E2, Estradiol; MII, Metaphase II; 2PN, 2 pronuclei; ET, Embryo transfer; FET, Frozen embryo transfer; SD, Standard deviation

A total of 166 ETs was performed, and the number of ET cycles per subject was 2.3 ± 1.25. Hormonally substituted FET was the major ET modality (92.8%), while modified natural FET was conducted in only 4.2%. A mean of 1.1 ± 0.31 embryos was transferred each time, and embryos were transferred mostly on day 3 of their developmental stage (91%).

### Pregnancy and Obstetric Outcomes

#### Pregnancy Outcomes

A total of 184 embryos were transferred, and the pregnancy outcomes were assessed (Table 3). The rates of pregnancy, clinical pregnancy, early miscarriage, and miscarriage were 28.6%, 19.2%, 28%, and 2.8%, respectively. The live birth rate was 52.3% per woman who underwent transfer and 18.2% per ET cycle. The potential factors entered as independent variables in the generalized linear mixed models for live births were the donor/recipient age, number of mature oocytes per recipient, sperm source, number of frozen embryos, and number of ETs. As shown in Table 4, the live birth rate significantly declined when the number of ETs was greater than one.

**Table 3:** Pregnancy outcomes of Israeli oocyte recipients who received oocytes from volunteer Israeli donors

Pregnancy rate per ET (%)	28.6
Clinical pregnancy rate per ET (%)	19.2
Early miscarriage rate per pregnancy (%)	28
Miscarriage rate per pregnancy (%)	2.8
Live birth rate per ET (%)	18.2

ET, Embryo transfer

**Table 4:** Multiple logistic regression analysis to estimate the variables that were significant in achieving live births among Israeli oocyte recipients

	Coefficient (B)	Sig	Odds (Exp(B))	95% C.I. for Odds	
Age of donor	−0.107	0.123	0.898	0.784	1.03
Age of recipient	−0.041	0.485	0.960	0.856	1.077
Source of sperm (partner vs donor)	0.03	0.943	1.031	0.443	2.399
Number of MII donated oocytes per recipient	0.024	0.655	1.025	0.920	1.141
Number of frozen embryos per recipient	−0.109	0.49	0.896	0.655	1.227
Number of ETs (1 vs > 1)	6.785	< 0.001	884.55	25.319	30,903.395

The association between the indicated parameters and the outcome of live births was assessed with the generalized linear mixed models which takes into account the repeated measures for donors. A *p* value of < 0.05 was considered significant

ET, Embryo transfer

#### Maternal and Neonatal Outcomes

Thirty-three pregnancies resulted in a live birth. Only one was a twin delivery. The obstetric outcomes are detailed in Table 5. The recorded maternal complications included HDP (n = 6, 18.2%) and GDM (n = 10, 32.3%). Most of the pregnancies were delivered by CS (n = 26, 78.8%). The neonatal outcomes of all 34 newborns were analyzed and pre-term delivery (n = 4, 12.1%), SGA (n = 4, 11.8%), LGA were observed (n = 4, 11.8%), 1-min Apgar ≤ 7 (n = 3, 8.8%), and 5-min Apgar ≤ 7 (n = 1, 2.9%) were observed. There were no very premature births (< 32 weeks’ gestation). The female/male ratio among the newborns was 1.125. Three infants have thus far been diagnosed with congenital malformations. One boy had been prenatally diagnosed with Klinefelter syndrome. One girl displayed signs of developmental delay and she was diagnosed with Waardenburg syndrome using exome sequencing. The third girl presented with cross ectopic kidney and developmental delay. No pathological findings were revealed in exome sequencing.

**Table 5:** Obstetric outcomes among Israeli recipients of oocytes from volunteer Israeli donors

Maternal	
HDP	6 (18.2)
*GDM*	
Diet regulated	7 (22.6)
Insulin treatment	3 (9.7)
*Mode of delivery*	
Vaginal	6 (18.2)
Instrumental vaginal	1 (3)
CS	26 (78.8)
Perinatal	
Preterm birth (> 32 and < 37 weeks)	4 (12.1)
*Birthweight (g)*	
AGA (> 10th and < 90th centile)	4 (11.8)
SGA (< 10th centile)	26 (76.4)
LGA (> 90th centile)	4 (11.8)
*Apgar score*	
≤ 7 at 1 min	3 (8.8)
≤ 7 at 5 min	1 (2.9)
*Sex*	
Female	18 (52.9)
Male	16 (47.1)
Congenital malformations	3 (8.8)

Values are presented as mean (standard deviation) or number (%)

HDP, Hypertensive disorder of pregnancy; GDM, Gestational diabetes mellitus; CS, Caesarian section; AGA, Appropriate for gestational age; SGA, Small for gestational age; LGA, Large for gestational age

## Discussion

In September 2010, Israeli law was amended to allow OD from compensated Israeli volunteers, thereby easing the limitations to ova donated by Israeli women undergoing fertility treatment (Levran et al., 1996; Rabinerson et al., 2002; Ministry of Health, State of Israel, 2005; Ministry of Health, State of Israel, 2010). To the best of our knowledge, this is the first study to characterize the Israeli donors and recipients and to characterize ova donation outcomes among them.

### Donors

The women who donated oocytes were mostly Ashkenazi Jewish, in their late twenties, single, childless, and employed. Almost all of them donated only once, all were financially compensated, and, as could be expected, all but one were Jewish (she defined herself as "without religion"). Judaism is the main religion in Israel (74.3%) and OD is a generally accepted practice (Schenker, 2013). Sunni Islam, the second most common religion in Israel (20.9%), absolutely forbids the use of third-party reproductive assistance (Chien, 2020). Oocyte donors have often been described as being young, well-educated, and, gainfully employed women similar to our donors, but, unlike our donors, they were often mothers who were altruistically motivated (Sauer et al., 1994; Söderström-Anttila, 1995; Klock et al., 1998; Byrd et al., 2002; Kenney & McGowan, 2010). In other studies, most of the donors were single and childless, and their main motive for donating oocytes was only monetary or both altruism and financial, but unlike our donors, they donated multiple times (Pennings et al., 2014; Rosenberg & Epstein, 1995; Tulay & Atılan, 2019). According to Pennings et al., the diversity of donor populations mainly reflects the differences in the countries’ legislation (Pennings et al., 2014). The Israeli law states that the oocyte donors must be unmarried women in order to prevent the possibility of the act being considered a form of mamzerut (Schenker, 2005; Ministry of Health, State of Israel, 2010). Additionally, the current law requires monetary compensation to cover the inconveniences that egg donors incur during the egg collection process, but not too high so that payment can become a major motive of women in financial difficulties (Ministry of Health, State of Israel, 2010). An additional interesting question is why most Israeli donors performed one treatment cycle, while in other studies, they donated multiple times. There are several possible reasons: (1) the low financial compensation. (2) difficulties related to the process, such as daily injections, vaginal ultrasounds, follow-ups, and surgical procedures under general anesthesia, etc. (3) doubts/regret about the decision to donate their oocytes (4) The appeal for donation is voluntary. We do not proactively ask the donor for additional cycles. Given the study's retrospective nature, we cannot answer this intriguing question, and further studies are needed.

### Recipients

All of the oocyte recipients in this study were Jewish and mostly in their mid-forties. They presented similar rates of chronic hypertension and DM type 2 as the same-aged women in the general population in Israel (Israel Center for Disease Control, 2017). Half of them were married and used their partner's semen, and the other half were unmarried and used donor semen. The main indication for using donated oocytes was a failure at IVF attempts that characterize women in their forties due to age-related decreased ovarian reserve. Although OD had originally been performed in patients with premature ovarian failure, it is now commonly used in perimenopausal/menopausal women as well (Sauer & Kavic, 2006). Over 50% of our oocyte recipients used sperm donors and all of them were unmarried. Similar figures have been reported from other parts of the world. The number of women receiving IVF cycles with both oocyte and sperm donation has grown globally in the last decade (Blázquez et al., 2016), mainly due to an increasing number of single women and same-sex couples (Human Fertilisation & Embryology Authority, 2019). As far as we know, there is no data regarding the prevalence of double gamete donation in Israel. There are several ethical challenges related to double gamete donation that should be considered (Huele et al., 2020): (1) the absolute absence of a genetic link between the (future) child and parent (s). (2) the potential existence of half genetically related siblings living elsewhere. (3) the creation of new surplus embryos in storage. (4) In the case of a single patient, the future child can be left a complete orphan if the mother dies.

### Ovarian Stimulation Outcomes

The ovarian stimulation outcomes among our oocyte donors, including the number of retrieved and mature oocytes and maturity rate, were excellent. These results were expected since the ovarian reserve of each oocyte donation candidate had been examined and only those with uncompromised ovarian reserve continued in the process. There was no incidence of ovarian hyperstimulation syndrome, most probably because the GnRH analog was used for triggering (Alama et al., 2013).

### Pregnancy Outcomes

At least one of the oocytes was fertilized among all the initiated cycles, yielding a high fertilization rate (~ 85%). ET was performed in 65 out of 69 recipients since four women decided not to undergo ET due to personal (non-medical) reasons. A total of 36 clinical pregnancies resulted, yielded an overall clinical pregnancy rate of 19.2% per ET, and a total of 34 (not including one ongoing pregnancy) live births resulted in an overall live birth rate of 18.2% per ET. Various rates of clinical pregnancies and live births have been published following egg donation. However, recent data are reported as being up to 60% clinical pregnancies per cycle and live birth rates as high as 50% per transfer (Malhotra et al., 2021; (SART) SFART. National summary report, 2020). Several reasons may underline the discrepancies between those rates and ours: (1) the oocytes of each of the donors were generally distributed to three recipients, depending on the total number of mature oocytes and the requirement of supplying at least four MII oocytes per recipient. We observed an average of 7.7 mature oocytes per recipient, resulting in an average of 4.6 frozen embryos per woman. These numbers are lower compared to other programs, which committed to a higher number of eggs or embryos. Limiting the number of eggs/embryos would inevitably negatively affect the pregnancy and live birth rates; (2) the median age of our recipients was 45 years, and about 10% of recipients were over 50 years, and several studies had found a negative association between advanced recipient age and pregnancy and live birth rates (Huang et al., 2008; Yeh et al., 2014). Huang et al. suggested that endometrial receptivity decreased with increased age (Huang et al., 2008); (3) ICSI was the only fertilization method in our study, and it had been shown that conventional IVF offers more embryo efficiency and increased implantation rate than ICSI in the presence of normal sperm and donor oocytes (Ten et al., 2022); (4) Our inclusion of a greater proportion of women who had repeatedly failed ET cycles may have included recipients with a missed uterine pathology that impaired their ability to conceive, and although the quality of the embryos is considered the main factor for implantation success, the uterine factor and endometrial receptivity must also be taken into account when pregnancy had not been achieved (Barri et al., 2014); (5) As dictated by national and international guidelines (Practice Committee of the American Society for Reproductive Medicine and the Practice Committee for the Society for Assisted Reproductive Technologies, 2021) only 10% of ETs involved the transfer of more than one embryo, and lower clinical pregnancy and birth live rates were observed in single ETs (SETs) versus double ETs (DETs) among oocyte recipients (Acharya et al., 2016; Klenov et al., 2018); (6) Only five ETs were fresh, and fresh ETs had been associated with better birth outcomes compared with frozen ones (Roeca et al., 2020). We preferred frozen ETs because of the time and logistics required to synchronize donor and recipient for a fresh ET, as well as the availability of excess embryos typically frozen after the initial fresh ET; (7) Most of the embryos in our study were transferred on day 3 of their developmental stage. An ET at the blastocyst stage is accompanied with a significant improvement in pregnancy rates compared to day-3 cleavage stage when the embryos are fresh (Insogna et al., 2021; Prapas et al., 2022) as well as vitrified (Kontopoulos et al., 2019). In our IVF unit, ET is not routinely performed in the blastocyst stage, and the day of ET (day-3 or −5) is determined on a case-by-case basis. In the OD program, ET is performed mainly on day-3 because of the small number of embryos per recipient. This small number increases the possibility that no embryos will be left for transfer on day-5. Therefore, we performed ET earlier, hoping that natural physiological conditions might benefit the embryo. ET on day-2 was done only in cases where day-3 was a Saturday, a day that that our IVF unit is closed. (8) Lastly, the Israeli regulations allow an unrestricted number of IVF treatments using the patients’ own oocytes until the age of 45. Many women continue performing hopeless treatment cycles that, according to the results of the current study, may lower their chance of succeeding in postponed OD treatment.

A review of pregnancy and birth rates in our IVF unit of infertile patients using their oocytes (same age range as the donors) presents comparable or even higher outcomes when compared to the current study where donors' oocytes were used [Hoffman et al., 2022., Levin et al., 2017., Bilgory et al., 2021). This comparison highlights the poor results of the Israeli OD program. Until recent months, we did not have imported oocyte donation programs in our unit. Therefore, we cannot compare OD programs regulated by Israeli law with those not which were carried out in the same unit. Such a comparison, most likely, would have strengthened our conclusions. The rate of other adverse pregnancy outcomes was low, probably because the oocytes were provided by young donors.

### Maternal Outcomes

In our study, most women using donor oocytes were over 40 years old, and with increasing age comes increasing risk for maternal and neonatal outcomes. Indeed, we observed high rates of maternal complications, including, HDP (~ 20%), GDM (~ 35%), and CS (~ 80%). Several systemic studies and reviews have suggested that IVF-ODs constitute an independent risk factor for maternal results when compared to both spontaneous pregnancies and pregnancies by IVF-autologous oocytes (Berntsen et al., 2021; Chih et al., 2021; Moreno-Sepulveda & Checa, 2019).

### Neonatal Outcomes

Reports on neonatal complications in IVF-OD have been conflicting (Savasi et al., 2016; Rizzello et al., 2020). In our study, the overall neonatal outcomes would appear to be reassuring, they were PTB ~ 12%, SGA ~ 12%, LGA ~ 12%, and an Apgar score ≤ 7 ~ 3%. Importantly, in the present study, all but one of the pregnancies were singletons, which significantly reduces the risks of adverse neonatal outcomes. In addition, the oocyte recipients who were older than 40 years of age were routinely referred to high-risk pregnancy clinicians. Although we do not have their detailed pregnancy surveillance findings, we are confident that appropriate preventive strategies (e.g., the use of low-dose aspirin) and appropriate screening (e.g., optimal glycemic control, treatment for anemia, etc.) were implemented.

Kamath and Sunkara observed no increased risk of congenital malformations following OD compared with autologous IVF births (Kamath and Sunkara, 2017). Similarly, three of the 34 children born via our OD program were subsequently discovered to have sustained them. One boy had been prenatally diagnosed with Klinefelter syndrome and the parents decided not to terminate the pregnancy. One girl displayed signs of developmental delay and she was diagnosed with Waardenburg syndrome using exome sequencing. Importantly, the mutation was discovered to be a de novo mutation. The third child was a girl who was diagnosed with cross ectopic kidney and developmental delay: exome sequencing failed to reveal any pathological findings. There is no association between OD and these malformations and a pre-OD evaluation of both the oocyte donor and the sperm source cannot prevent these specific ones.

### Comparison with Other Egg Donation Programs

It is important to note that we did not show a direct correlation between low pregnancy and live birth rates and the legal restrictions. However, there is no doubt that those are lower than published values in other OD programs, with whom the incidence of live birth is as high as 50% per cycle (Malhotra et al., 2021; (SART) SFART. National summary report, 2020; Martínez et al., 2020). A matched control group could have strengthened our conclusions. However, other OD programs guarantee more oocytes and even blastocysts raising the pregnancy and live birth rates. Therefore, finding a matched control group from elsewhere is very problematic. We concur with the statement that donor and recipient factors affect pregnancy and live birth rates in oocyte donor cycles. Ideal donors fall in the age range of mid 20s or early 30s. A donor younger than 25 does not predict more favorable outcomes (Humphries et al., 2019), while donors older than 35 have comparatively reduced live birth rates than their younger counterparts (Pennings et al., 2014). The mean age of our donors was 29. Apart from age, other donor factors such as general health, BMI, ovarian reserve markers including anti-Mullerian hormone (AMH), FSH, and AFC, as well as the number of oocytes retrieved have also been studied, with variable association with OD-IVF outcome. In our IVF unit, the selection criteria of recruited donors are strict regarding their general health, BMI, and ovarian reserve markers.

The relationship between OD outcomes and uterine receptivity in older oocyte-recipient women is controversial. Some found OD outcomes declined in recipients > 45 years and declined further in recipients > 50 years (Soares et al., 2005). The mean age of our recipients was 44.6, and it might have negatively affected pregnancy rates and live births. However, this factor alone cannot explain the meager rates found in our study.

The legal restrictions in Israel require us to take actions that have previously been proven to negatively affect the pregnancy and live birth rates in OD cycles:

1. Our IVF unit benefits financially only if the oocytes of one donor are distributed to 3 recipients. Therefore, each recipient receives a small number of eggs and finishes with a low number of embryos.
2. ET is performed on day three due to the low number of embryos per recipient.
3. We commit to mature eggs, as only a mature egg has the potential to be fertilized, and therefore ICSI is the only fertilization method we use. This is not a requirement of the Israeli law, but a medical decision of our unit. Being that the law limits the ED procedure in many aspects, we take this step in order to maximize the potential for fertilization.
4. According to the law, the coupling between a donor and the recipients requires approval of the MOH before the donation. This creates difficulty in synchronizing, and therefore the embryos are cryopreserved and transferred later.

We strongly believe that it is essential to highlight, for the first time, the possible relation between the treatment strategies we adopted following the legal restrictions and the low outcomes in the Israeli OD program.

### Limitations

This study has several other limitations that bear mention. Firstly, its retrospective design limits our ability to obtain more details about the attitudes, motivations, knowledge, and expectations of young Israeli women with regard to OD. The influence of these factors warrants a prospective study using standardized questionnaires or interviews for establishing a recruitment process that will increase the number of Israeli donors. Secondly, the study included a small sample size, which could compromise the strength of the conclusions, thus calling for further studies on larger populations. Thirdly, the study was conducted in a single institution located in the center of Tel Aviv, which is the most liberal area in Israel, a factor that may influence the donors and recipients’ characterizations. However, given that few Israeli centers perform ODs according to Israeli law, women arrive from throughout the country and very likely reflect this population nationwide.

### Policy Implications

The compliance of young Israeli women in donating their oocytes is extremely low. There was an average of five donors per year despite the high demand from infertile Israeli women and in contrast to the global trend towards an increase in the number of egg donors ((SART) SFART. National summary report, 2020; Nyboe Andersen et al., 2009; Geyter et al., 2020; Kawwass et al., 2013). Several reasons may be responsible for the reluctance to donate oocytes: (1) religious, cultural, and social norms in Israel do not encourage oocyte donation; (2) lack of knowledge about the process; (3) need for a considerable investment of time to carry out the preparations; (4) concerns about the process (e.g., side effects and complications); (5) compensation for their time and effort considered inadequate by potential donors; (6) lack of a recruitment program to inform and encourage potential donors. The current study did not examine the last issue, and further studies are needed to explore attitudes, motivations, knowledge, and decision-making processes of young Israeli women with regard to oocyte donation. The provisions of the Israeli law (Schenker, 2005; Ministry of Health, State of Israel, 2010) also constitute a limiting factor for the recruitment of oocyte donors. For example, the oocyte donors must be unmarried in order to prevent the possibility of the act being considered a form of mamzerut. The percentage of Israeli women in the donor age group that are unmarried: 20–24 years: 74.8; 25–29 years: 44.3; 30–34 years: 27.5 (Israel Central Bureau of Statistics, 2021). Most of the donors in our unit were in their late twenties-early thirties, and in this age group, most women in Israel are married. Therefore, if the law allowed egg donations from married women, the pool of donated eggs would substantially increase. In Addition, The Law of Return is an Israeli law that gives Jews, people with one or more Jewish grandparent, and their spouses the right to relocate to Israel and acquire an Israeli citizenship. If a person has a Jewish grandparent but their mother is not Jewish, they will not be considered a Jew according to religious law but are entitled to an Israeli citizenship. Thus, thousands of women in Israel are eligible for citizenship but according to the religious law are not Jewish. These women are considered irreligious, and since they are not Jewish, they cannot donate eggs to Jewish women. Amending the ED law in a way that allows women of this status to donate eggs to Jewish women would substantially increase the pool of donated eggs in Israel. Additionally, the law requires the inclusion of the donors’ details in the MOH’s database and Newborn Registry, which may arouse concerns among donors. Furthermore, donors undergo a complex process that includes medical examinations, psychological assessments, and approval by a number of committees. Finally, the monetary compensation is so low that egg donations are clearly a matter of compassion and generosity. We believe that easing some if not all of these restrictions will increase the number of oocyte donors and provide the opportunity for women to realize their hopes to bear children.

## Conclusions

This study is the first to characterize the Israeli volunteer oocyte donors and their recipients and to determine the Israeli OD program outcomes. The principal findings of this study were that an extremely low number of young Israeli potential donors participate in this program, and that there is a considerable uniformity among them. Liberalizing the very stringent laws regulating OD may go far in enhancing the likelihood of greater success of OD in Israel. Although one-half of our OD recipients completed the process with a child, pregnancy and live birth rates were lower than expected, which underscores the need for improvements in technique, including more oocytes available to each recipient, conventional IVF, fresh ET, a day-5 ET, and more. Obstetric outcomes were generally favorable, although there was a higher incidence of maternal complications, as could be expected in women of advanced age, therefore calling for targeted pre-pregnancy care and close surveillance during pregnancy.

## Data Availability

Data are available upon request.

## Abbreviations

OD: Oocyte donation
MOH: Ministry of Health
IVF: In vitro fertilization
AFC: Antral follicle count
FSH: Follicle-stimulating hormone
ET: Embryo transfer
BMI: Body mass index
TSH: Thyroid-stimulating hormone
ART: Assisted reproductive technology
OPU: Ovum pick-up
MII: Metaphase II
2PNs: 2 Pronuclei
HDP: Disorders of pregnancy
PIH: Pregnancy-induced hypertension
GDM: Gestational diabetes mellitus
CS: Caesarean section
PTB: Preterm birth
LGA: Large for gestational age
SGA: Small for gestational age
AGA: Appropriate for gestational age
GnRH: Gonadotropin releasing hormone
ICSI: Intracytoplasmic sperm injection
FET: Frozen ET
SD: Standard deviation
SET: Single ET
DET: Double ET
